# Metformin therapy and the risk of colorectal adenoma in patients with type 2 diabetes: A meta-analysis

**DOI:** 10.18632/oncotarget.13633

**Published:** 2016-11-26

**Authors:** Yi-Chao Hou, Qiang Hu, Jiao Huang, Jing-Yuan Fang, Hua Xiong

**Affiliations:** ^1^ Division of Gastroenterology and Hepatology, Key Laboratory Gastroenterology and Hepatology, Ministry of Health, State Key Laboratory for Oncogenes and Related Genes, Renji Hospital, School of Medicine, Shanghai Jiao Tong University, Shanghai Institute of Digestive Disease, Shanghai 200001, China

**Keywords:** metformin, colorectal adenoma, advanced adenoma, diabetes, meta-analysis

## Abstract

**Background:**

Existing data evaluating the impact of metformin on the colorectal adenoma (CRA) risk in patients suffering from type 2 diabetes (T2D) are limited and controversial. We therefore summarized the studies currently available and assessed the relationship between metformin treatment and risk of CRA in T2D patients.

**Methods:**

We systematically searched databases for eligible studies that explored the impact of metformin treatment on the occurrence of CRA in T2D patients from inception to June 2016. The summary odds ratio (OR) estimates with their 95% confidence interval (CI) were derived using random-effect, generic inverse variance methods. Sensitivity analysis and subgroup analysis were performed.

**Results:**

Seven studies involving 7178 participants met the inclusion criteria. The pooling showed that metformin therapy has a 27% decrease in the CRA risk (OR, 0.73; 95% CI, 0.58 - 0.90). In subgroup analysis, we detected that metformin exhibits significant chemoprevention effects in Asia region (OR, 0.68; 95% CI, 0.48 - 0.96). Similar results were identified in both studies with adjusted ORs and high-quality studies (OR, 0.66; 95% CI, 0.50 - 0.86 and OR, 0.70; 95% CI, 0.58 - 0.84, respectively). Of note, an inverse relationship was noted that metformin therapy may result in a significant decrease in the advanced adenoma risk (OR, 0.52; 95% CI, 0.38 - 0.72). Low heterogeneity was observed, however, the results remained robust in multiplesensitivity analyses.

**Conclusions:**

This meta-analysis indicates that metformin therapy is correlated with a significant decrease in the risk of CRA and advanced adenoma in T2D patients. Further confirmatory studies are warranted.

Existing data evaluating the impact of metformin on the colorectal adenoma (CRA) risk in patients suffering from type 2 diabetes (T2D) are limited and controversial. We therefore summarized the studies currently available and assessed the relationship between metformin treatment and risk of CRA in T2D patients.

We systematically searched databases for eligible studies that explored the impact of metformin treatment on the occurrence of CRA in T2D patients from inception to June 2016. The summary odds ratio (OR) estimates with their 95% confidence interval (CI) were derived using random-effect, generic inverse variance methods. Sensitivity analysis and subgroup analysis were performed.

Seven studies involving 7178 participants met the inclusion criteria. The pooling showed that metformin therapy has a 27% decrease in the CRA risk (OR, 0.73; 95% CI, 0.58 – 0.90). In subgroup analysis, we detected that metformin exhibits significant chemoprevention effects in Asia region (OR, 0.68; 95% CI, 0.48 – 0.96). Similar results were identified in both studies with adjusted ORs and high-quality studies (OR, 0.66; 95% CI, 0.50 – 0.86 and OR, 0.70; 95% CI, 0.58 – 0.84, respectively). Of note, an inverse relationship was noted that metformin therapy may result in a significant decrease in the advanced adenoma risk (OR, 0.52; 95% CI, 0.38 – 0.72). Low heterogeneity was observed, however, the results remained robust in multiple sensitivity analyses.

This meta-analysis indicates that metformin therapy is correlated with a significant decrease in the risk of CRA and advanced adenoma in T2D patients. Further confirmatory studies are warranted.

## INTRODUCTION

Colorectal cancer (CRC) represents the fourth most common cause of cancer death worldwide [1], with a continuous increase in prevalence and mortality [2]. Based on the widely recognized adenoma-carcinomas sequence, most CRCs originate as precancerous adenomas [3]. Therefore, the removal of colorectal adenoma (CRA) may reduce the probability of subsequent occurrence of advanced adenoma and CRC [4].

In recent years, however, a conversion in approach from early detection of precancerous adenoma and cancer to new cancer prevention strategies, such as chemoprevention, which may be beneficial for optimal CRC prevention in average-risk populations when combined with colonoscopies has been studied [5]. Many agents are explored extensively for chemopreventive potential against CRC development, such as calcium [6], folic acid [7], vitamin [8] and nonsteroidal anti-inflammatory drugs (NSAIDs) [9]. Among these pharmacological agents available for prophylaxis, NSAIDs, particularly COX-2 inhibitors, have exhibited the most promising chemopreventive effect on decreasing the risk of CRC [10]. However, COX-2 inhibitors have some serious side effects, including gastrointestinal bleeding, ischemic stroke and cardiovascular events [11, 12], and other agents that originally showed promise in this setting no longer demonstrate any significant efficacy. Therefore, novel drugs with high safety and effectiveness in the prevention of CRC are urgently required to be developed. A growing body of studies have suggested that CRC is correlated with several lifestyle diseases, including obesity and diabetes mellitus [13–15]. Thus, it has suggested that these conditions may be the targets for prevention of CRC.

Globally, diabetes is a well-established, independent risk factor for CRC; hyperinsulinemia induced by insulin resistance has been considered as an importantly latent mechanism linking obesity, immobile life and poor diet to CRC [16–18]. Encouragingly, several preclinical studies have shown that conventional antidiabetic medication, especially metformin, may modify the risk of CRC. Metformin is a biguanide derivative that is frequently used as the first-line drug for type 2 diabetes (T2D) [19]. Metformin improves insulin resistance and lowers glucose production by inhibiting gluconeogenesis and glycogen decomposition in the liver and increasing glucose absorption by muscle tissues [20, 21]. Unlike other oral antidiabetic drugs, metformin does not stimulate insulin secretion directly and has been recommended as the initial treatment of diabetes [22]. Importantly, metformin is well tolerated in most subjects with a good safety profile and low cost, thereby being easily accessible in clinical practice for large populations. Experimental data obtained from in vitro and animal studies identified that metformin can suppress proliferation of cultured cancer cells and lower cancer risk [23]. In addition, previous studies have indicated that metformin is involved in the tumor-suppressor pathway by inhibiting lipogenic pathways and stimulating liver kinase B1 (LKB1)-dependent activation of 5-AMP-activated protein kinase (AMPK), an inhibitor of cell proliferation via the mammalian target of rapamycin (mTOR) pathway [24, 25]. Similarly, in vitro experiments have shown that metformin triggers cell growth arrest [26], induces cell apoptosis [27], and inhibits invasive properties of cancer cells [28]. Animal experiments concur with these findings. Non-diabetic mouse model has shown that metformin suppresses the polyp growth [29] and colonic epithelial proliferation [30], suggesting a promising candidate for the chemoprevention against CRC. In addition, a clinical trial in humans showed that oral administration of metformin at a low-dose of 250 mg/day is safe and able to inhibit the colorectal aberrant crypt foci (ACF) formation [31]. ACF has been regarded as a very useful CRC biomarker [32].

However, existing data evaluating the association between metformin and CRA are limited and controversial. Thus, a meta-analysis was performed in this paper to summarize literature available to date to assess the impact of metformin treatment on the CRA risk in patients suffering from T2D.

## RESULTS

### Search results

**Figure 1:**
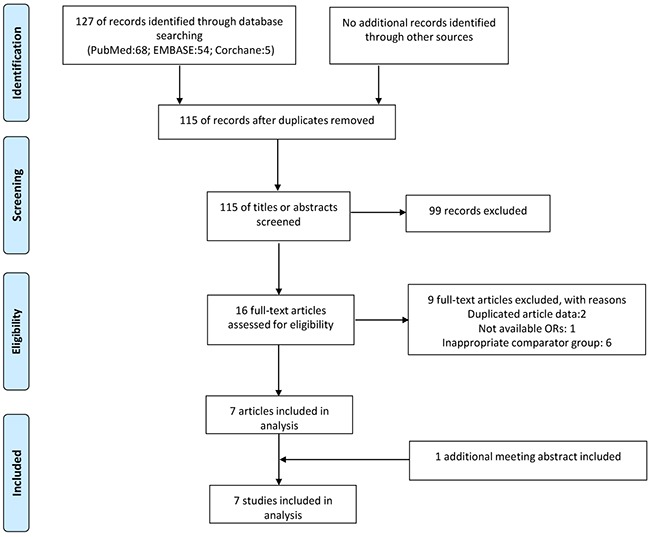
Flow diagram of included and excluded trials

A flowchart describing the identification and selection of studies is summarized in Figure 1. A total of 127 articles were obtained until June 2016 by searching the databases systemically. The references of the included studies and relevant reviews did not yield additional studies. After exclusion of duplicates and screening the title and abstracts, the full texts of 16 articles remained for further evaluation. After reviewing the full text, 7 observational studies assessing the relationship between metformin therapy and CRA risk in T2D patients were selected for eligibility [33–39]. One meeting abstract and an article were found, both of which were based on overlapping data from the same cohort study [33, 40]. To ensure no duplicate data were included, only the largest sample size or the most rational information from retrieved studies was selected [40].

### Study characteristics

A total of seven eligible retrospective studies involving 7178 patients, including 2660 cases and 4518 controls were included in this analysis. Among them, four studies represented Western population (Unites States; ref. [34, 35, 39, 40]), the remaining three studies were performed in an Asian population (Korea; ref. [36–38]). Chronologically, the earliest study started in 1998, while the most recent one ended in 2014. Six selected studies were published between 2008 and 2015. Seven studies investigated the relationship between metformin treatment and the risk of CRA in T2D patients, of which five studies performed adjusted odds ratio (OR) for confounders [35–39]. Five studies adjusted for following confounders: age (5/5), gender (5/5), BMI (4/5), aspirin use (4/5), smoking status (3/5), insulin use (2/5), alcohol use (2/5). In addition, four studies reported the relationship between metformin treatment and the advanced adenoma risk [37, 38, 40, 41]. Of these four studies, only two studies adjusted for relevant confounders. The properties of each study included in the meta-analysis can be found in Table 1. Using the Newcastle-Ottawa quality scale (NOS) tool [42], the average score was 7.1, and the score for each study was 6 or above, suggesting all studies except two were of high quality. Quality assessments of each included study are presented in Supplementary Table S1.

**Table 1:** Main characteristics of studies included in the meta-analysis of association between metformin therapy and risk of CRA in T2D patients

Source^a^	Recruitment period	Study type	Gender (% male)	Age (mean ± SD)	Total participants	OR (95% CI)	Controlled variables	NOS scores
Chung, 2008, Korea	2003-2006	Case-control	I:52 C:52	I: 66.8 ± 9.4 C: 66.2 ± 10.9	100	A: 0.70 (0.30-1.40)	Age, gender, BMI, duration of DM, serum levels of HbA1c and lipids, use of insulin and aspirin	8
Lee, 2012, Korea	1998-2008	Cohort	I: 71 R:72	I: 61.4 ± 8.0 C: 62.3 ± 7.9	240	A: 0.27 (0.10-0.75)	Age, gender, BMI, stage of cancer, family history of CRC, follow-up duration, No. of total colonoscopies, interval to first follow-up colonoscopy, No. of baseline CRA, treatment modality, use of aspirin, insulin and thiazolidinediones	6
Kanadiya, 2013, U.S.	2008-2009	Cohort	NA	NA	405	A: 0.55 (0.34-0.87)	Age, gender, smoking status, use of alcohol	8
Jain, 2014, U.S.	2012-2014	Cohort	NA	NA	676	C: 0.68 (0.48-0.95)	NA	7
Cho, 2014, Korea	2001-2013	Cohort	I: 60 R: 54	I: 60.1 ± 10.8 C: 63.9 ± 12.0	3105	A: 0.73 (0.55-0.98)	Age, gender, BMI, triglyceride, HbA1c, duration of DM, smoking status, use of aspirin and stain	8
Kim, 2015, Korea	2002-2012	Cohort	I: 70 R: 67	I: 58.8 ± 9.9 C: 61.6 ± 10.3	240	A: 0.87 (0.45-1.62)	Age, gender, BMI, smoking status, use of aspirin and alcohol	7
Marks, 2015, U.S.	2000-2009	Cohort	I: 61 R: 64	NA	2412	C: 0.94 (0.80-1.11)	NA	6

^a^First author, publication year, country.

I, intervention group; R, reference group; NA, not available; OR, odds ratio; 95% CI, 95% confidence interval; A, adjusted effect size; C, computed effect size; BMI, body mass index; CRC, colorectal cancer; CRA, colorectal adenoma; T2D, type 2 diabetes; DM, Diabetes mellitus; NOS, Newcastle-Ottawa quality scale.

### Overall CRA

**Figure 2:**
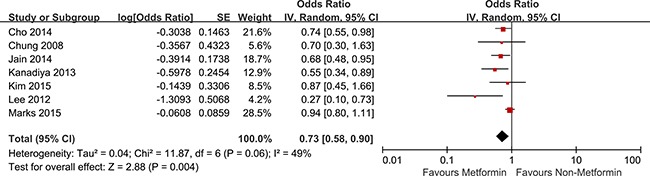
Forest plot for the meta-analysis of studies examining the association between metformin treatment and risk of CRA in T2D patients CI, confidence interval; IV, inverse variance; SE, standard error; CRA, colorectal adenoma; T2D, type 2 diabetes.

Seven studies with available data were included for quantitative meta-analysis [34–40]. Among the seven observational studies that reported CRA incidence, four studies demonstrated an apparent chemoprotective association and the remaining three studies did not indicate any statistically significant relationship. In a pooled analysis of all seven studies, compared with treatment without metformin, treatment with metformin was correlated significantly with the reduction in CRA incidence by 27% (OR, 0.73; 95% CI, 0.58 – 0.90; *P* <0.01), with a low heterogeneity (I^2^ = 49%; *Q* = 11.87, *P* = 0.06) (Figure 2).

### Colorectal advanced adenoma

**Figure 3:**
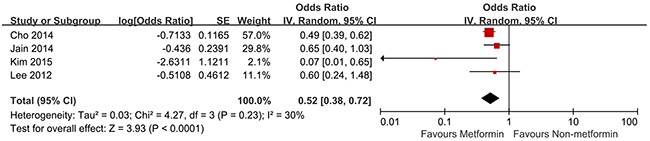
Forest plot for the meta-analysis of studies examining the association between metformin treatment and risk of advanced adenoma in T2D patients CI, confidence interval; IV, inverse variance; SE, standard error; T2D, type 2 diabetes.

Only four studies reported the incidence of colorectal advanced adenoma in T2D patients with metformin therapy [36–38, 40]. Of these four studies, two studies showed an apparent chemoprotective association. In contract, the other two studies did not show statistical differences between metformin therapy group and non-metformin therapy group. Pooling showed that there was statistically significant association between metformin therapy and colorectal advanced adenoma (OR, 0.52; 95% CI, 0.38 – 0.72; *P* <0.01), with insignificant heterogeneity (I^2^ = 30%, *Q* = 4.27, *P* = 0.23) (Figure 3).

### Subgroup analyses

Subgroup analyses across several different variables were further performed to investigate the heterogeneity of the studies (Table 2). A reduction with statistical significance in CRA risk in T2D patients treated with metformin was obtained in studies conducted in Korea region or studies with adjusted ORs (OR, 0.68; 95% CI, 0.48-0.96; *P* = 0.03 and OR, 0.66; 95% CI, 0.50 – 0.86; *P* <0.01, respectively), which were consistent with the result of the overall analysis. In contrast, a borderline association was noted among the three studies conducted in the United States and studies with unadjusted ORs (OR, 0.74; 95% CI, 0.54 – 1.03; *P* = 0.07 and OR, 0.83; 95% CI, 0.60 – 1.13; *P* = 0.24, respectively). In the subgroup analyses regarding study quality, high-quality studies revealed an inverse relationship between metformin therapy and CRA risk in T2D patients (OR, 0.70; 95% CI, 0.58 – 0.84; *P* <0.01), with null heterogeneity (I^2^= 0%; *Q* = 1.55, *P* = 0.82). However, there was a statistically insignificant association in low-quality group (OR, 0.56; 95% CI, 0.17 – 1.86; *P* = 0.34).

**Table 2:** Subgroup analyses of relationship between metformin therapy and risk of CRA in T2D patients

			Test of Relationship		Test of Heterogeneity		
**Subgroup**	**No. of studies**	**No. of patients**	**OR (95% CI)**	***P*** **value**	***Q*** **value**	***P*** **value**	**I^2^, %**
Overall	7	7178	0.73 (0.58-0.90)	<0.01	11.75	0.06	49
Study location							
Asia	4	3685	0.68 (0.48-0.96)	0.03	4.07	0.25	26
North America	3	3493	0.74 (0.54-1.03)	0.07	6.28	0.04	68
Study quality							
High (NOS score>6)	5	4286	0.70 (0.58-0.84)	<0.01	1.55	0.82	0
Low (NOS score≤6)	2	2892	0.56 (0.17-1.86)	0.34	5.90	0.02	83
Adjusted confounders							
Yes	5	4090	0.66 (0.50-0.86)	<0.01	4.91	0.30	19
No	2	3088	0.83 (0.60-1.13)	0.24	2.91	0.09	66

OR, odds ratio; 95%CI, 95% confidence interval; NOS, Newcastle-Ottawa quality assessment scale; CRA, colorectal adenoma; T2D, type 2 diabetes.

### Sensitivity analysis

Multiple sensitivity analyses were performed to further confirm the validity of the results. It was found that none of the exclusions of a specific study would change the magnitude or direction of the summary effect for the correlation between metformin therapy and CRA risk in T2D patients (OR, 0.67 – 0.78, *P* <0.05). Additionally, the significant association remained unchanged after excluding one study [38] reported with **relative risk (RR)** or one study [40] published in abstract form (OR, 0.65; 95% CI, 0.53 – 0.80; *P* <0.01 and OR, 0.66; 95% CI, 0.50 – 0.86; *P* <0.01, respectively). Furthermore, we also evaluated the effect of the large included studies on overall pooled estimate. When removing two large included studies [34, 37], the comparison remained unchanged and still statistically significant (OR, 0.63; 95% CI, 0.49 – 0.81; *P* <0.01).

### Publication bias

No evidence supporting publication bias was found based on visual inspection of the funnel plot and Begg test (*P* = 0.368) (Supplementary Figure S1).

## DISCUSSION

This represents the most contemporary meta-analysis, to the best of our knowledge, demonstrating an association between metformin therapy and CRA risk in T2D patients. The findings of the current study demonstrated that metformin therapy was reversely correlated with the decrease in the risk of CRA in T2D patients, with an about 27% reduction compared with the non-metformin therapy. Moreover, we identified for the first time that there was a statistical significant inverse relationship between metformin therapy and the colorectal advanced adenoma risk in T2D patients. No substantial difference in pooled estimates was detected in multiple sensitivity analyses, and no individual study had excessive influence on the overall results. Our findings were similar to a recent meta-analysis [43] published on this topic; however, the authors did not include a case-control study [39] met the prespecified inclusion criteria. It should be noted that this meta-analysis included only three studies exploring the effect of metformin on the risk of CRA, comprising 3745 subjects. Our meta-analysis had a markedly larger sample size than this study; we also evaluated the advanced adenoma risk with metformin therapy, and our study was up to date.

In the subgroup analyses, the results were significantly affected by study location, ORs with or without adjusted confounders, and study quality. When stratifying studies by location, it was demonstrated that metformin therapy was associated with a significant decrease in CRA risk in Asia, while no significant association was found in North America. Of interest, the antineoplastic relationship between metformin therapy and CRA risk was more evident in Asian people. Differences observed in the Asian and Western population in this meta-analysis may be due to differences in dietary habits and/or other cultural behaviors. For example, red meat intake or milk products are more popular in western countries than in Asia, which may be strong risk factors for CRA incidence [44, 45]. Another subgroup analysis showed that studies with adjusted confounders, including age, gender, BMI, smoking status, use of aspirin, insulin and alcohol, all of which are known to confound CRA incidence, had a stronger inverse association than those without adjusted confounders, indicating that the above mentioned factors may also contribute to the heterogeneity to some extent. When we stratified the studies by study quality, it was found that high-quality studies showed a significantly inverse association, however, no significant association was detected in studies with low quality. We believe that the logical outcome of subgroups of studies with adjusted confounders and high-quality can more accurately reflect the real effects.

Evidence has suggested that T2D patients have a higher risk of colon adenoma [46]. While the mechanisms are probably manifold and not yet completely clarified, insulin resistance, which results in subsequent hyperinsulinemia and hyperglycemia, is the most commonly proposed scenario, since insulin is able to promote mitosis through specific, high-affinity binding to the IGF-1 receptor [46–48]. A relationship between increased levels of circulating insulin and cancer has been investigated, and the results indicated that cancer growth may be affected by IGF-1 signaling axis [49]. Moreover, insulin is able to directly stimulate cell proliferation by binding to its cognate receptor, insulin receptor, or IGF-1 receptor, thereby activating multiple signaling pathways [50, 51]. However, metformin therapy decreases levels of IGF-1, improves insulin resistance in peripheral tissues, and alleviates the circulating insulin levels, which, in turn, may result in reduced risk of cancer [52, 53]. In addition to the improvement of insulin sensitivity, the promotion of weight loss, and other systematic effects, metformin has tumor-cell specific effects [54]. It has been well established in experimental studies that metformin exerts the anti-cancer activity through activation of AMPK and subsequent inhibition of the mTOR pathway, which is a downstream effector of growth factor signaling and is frequently activated in cancer cells [55]. Furthermore, in vitro studies have shown that metformin can also induce cell cycle arrest and induce cell apoptosis by inhibiting the expression of cyclin D1 and phosphorylation of retinoblastoma tumor suppressor protein (Rb) [56]. In vivo studies have demonstrated the direct chemopreventive effect of metformin in the APC^Min/+^ mice, a mouse model of familial adenomatous polyposis [29]. In a different mouse model of colon carcinoma, it has been found that metformin is able to delay the onset of P53-dificient colon cancer in mice [57]. Moreover, metformin has been shown to suppress azoxymethane-induced formation of ACF, a reliable surrogate biomarker of CRC, via the inhibition of mTOR pathway by activating AMPK [30, 32]. Other possible underlying mechanisms of the antineoplastic potential of metformin include its obesity antagonizing action [58], anti-inflammatory effect [59], and cancer stem cells killing effect [60]. These data strongly support the notion that metformin is one of the promising candidates for cancer therapeutics. However, the underlying mechanisms are eagerly awaited to be investigated through further detailed studies.

The main strength of the present meta-analysis is that by including only observational studies we were able to conduct an objective analysis on a large number of participants over a long follow-up period. In order to minimize the impact of confounding, the estimates from fully adjusted multivariable models were employed for the most of studies included. Both subgroup analyses and sensitivity analysis were conducted to explore the possible sources of heterogeneity. In addition, this study was performed based on prespecified selection criteria and a systematic search of the literature. Furthermore, our meta-analysis suggested that metformin may play a significant role in CRC chemoprevention by acting at early stages of adenoma onset and advancement in the adenoma-carcinoma sequence.

However, caution is required when interpreting these results. Limitations of our meta-analysis in general are that the validity is dependent on inherent limitations of observational studies on adjustment of confounding factors. All of the selected studies were observational studies (cohort and case-control) in which the information on metformin use was obtained retrospectively. Observational studies have methodical shortcomings and are prone to time-related biases, such as immortal time bias and time-lagging issues [61]. Additionally, in cohort studies there is a higher risk of indelible bias, mainly confounding. Although five out of seven studies adjusted for different confounders, the other two studies provided the unadjusted ORs, which may still influence the result and limiting comparability. The definitions of metformin use were different across the included studies, which may also lead to heterogeneity. Historical medical data, such as exact dose and duration of metformin use, and other adjunctive therapies, were incomplete. Hence, neither dose-response nor duration-response association between metformin therapy and risk of CRA in T2D patients could be established. We could not obtain data about history of exposure to tobacco smoke and alcohol consumption, which are strong risk factors for CRA [62, 63]. In this paper, metformin treatment was compared with thiazolidinediones, sulfonylureas, insulin, or other oral antidiabetic drugs, all of which may also affect the reliability of the results of the meta-analysis. It should be noted that thiazolidinediones, insulin, and other hypoglycemic drugs are able to affect the development of CRA [64, 65]. Therefore, it could be speculated that the observed useful activities of metformin might be an overestimation partially because of the adenoma-modifying activity resulted from other hypoglycemic drugs in the control group. Additionally, the conclusions regarding metformin therapy and the risk of advanced adenoma may be limited due to the inadequate sample size, and need to be further investigated. Finally, even though no conclusive evidence supporting publication bias was observed via visual examination of funnel plot and the Begg test, it is difficult to rule out completely the probability of publication bias since only seven articles were selected in the quantitative meta-analysis.

In conclusion, the results of this paper demonstrated that in T2D patients treatment with metformin is likely correlated with a significant decrease in both CRA risk and advanced adenoma risk. Considering the low side effect of metformin and the rising incidence rate of CRA, this study may provide significant public health implication for the prevention of CRC through decreasing the incidence of CRA. However, further studies, especially well-designed randomized, controlled trials, are awaited to substantiate these benefits from early observational studies.

## MATERIALS AND METHODS

### Literature search

The present meta-analysis was implemented according to the Preferred Reporting Items for Systematic Reviews and Meta-Analyses (PRISMA) guideline [66] and the epidemiological guidelines for meta-analysis of observational epidemiological studies [67]. The PubMed, Embase and the Cochrane Library were searched systematically to include studies until June 2016. The suitable key terms and/or corresponding text terms were employed: (“colorectal neoplasms” OR “colon neoplasm*” OR “colon polyp*” OR “colon adenoma*” OR “large bowel neoplasm*” OR “large bowel polyp*” OR “large bowel adenoma*” OR “rectum neoplasm*” OR “rectum polyp*” OR “rectum adenoma*”) AND (“metformin” OR “dimethylbiguanidine” OR “dimethylguanylguanidine” OR “glucophage” OR “metformin hydrochloride” OR “hydrochloride, metformin” OR “metformin HCl” OR “HCl, metformin”). Details of the search description for PubMed can be found in Supplementary Table S2. The reference lists of the retrieved full-text publications and reviews published on this relevant topic were hand-checked and authors contacted for further information where necessary. Additionally, we also inspected meeting abstracts from major gastroenterology conferences (including Digestive Disease Week, Canadian Digestive Disease Week, United European Gastroenterology Week, American College of Gastroenterology and the Asia-Pacific Digestive Week) for the presence of unpublished work on the relevant topic in the past five years. When multiple publications from an identical study were detected, the publication that was more informative was selected.

### Eligibility criteria

The observational studies were integrated due to the absence of available dates from randomized clinical trials. Eligible studies with the following criteria were included in this analysis: 1) the relationship between metformin therapy and CRA was evaluated; 2) metformin therapy was compared with non-metformin therapy; 3) OR or RR with the corresponding 95% CIs reported or data were provided for their calculation; 4) T2D was identified prior to CRA based on medical or pathological diagnosis; 5) the papers were published in English. All studies with the following conditions were excluded from the analysis—the subjects enrolled in the studies were type 1 diabetes patients, or patients with a history of familial adenomatous polyposis, inflammatory bowel disease, or cancers other than CRC. Studies in which other form of hypoglycemic drugs were compared with reference therapy or the risk of CRC was reported with the use of metformin were also excluded.

### Data extraction and quality assessment

Characteristics of retrieved reports were extracted by two members independently by scanning the titles and abstracts. The full text of potentially relevant articles were further evaluated. For those studies that met the inclusion criteria as mentioned above, two members extracted data independently according to the prespecified selection criteria. Any disagreements were resolved by discussion to reach a consensus. The extracted data from each study included the first author's last name, publication date (year), the site where the study was performed, recruitment period, study type, sex ratio, age, the number of total participants, reported or computed ORs, confounding adjustment and quality scores. Whenever possible, the adjusted estimates were summarized from the original article; alternatively the unadjusted ORs were calculated from studies containing raw data. In order to better identify the risk of bias between-study groups, quality assessment was conducted in observational studies using the **NOS quality tool**. The NOS comprises three categories: selection, comparability, and ascertainment of outcome. Scores range from 0 to 9, and studies with a score of more than or equal to 7 were classified as high quality studies, while studies with a score of less than or equal to 6 were considered low quality studies.

### Statistical analysis

All analyses were carried out based on the guidelines referenced in the Cochrane Handbook for Systematic Reviews of Interventions (version 5.1.0) [68]. OR was used to measure the effect size for original articles using incident cases of CRAs as the primary outcome and advanced adenoma as the secondary outcome. The point estimates and standard errors were pooled from individual studies with the inverse variance method,[69] in which the weight of each article was assigned on the basis of its variance. A model of random-effect was employed to analyze the data independent of heterogeneity, which renders more precise and authentic results [69]. The heterogeneity between studies was evaluated by calculating the Cochran’ *Q* statistic with a significance level of *P* < 0.10 [70]. Visual observation of the forest plots was used to identify the statistic heterogeneity, which was further complemented by the I^2^ statistic, a test used to quantify inconsistency across studies resulted from heterogeneity rather than from chance. A value of I^2^ of 0%–30%, 31%–50%, 51%–75%, and 75%–100% each represents an insignificant, low, moderate, and considerable heterogeneity, respectively [71].

Between-study sources of heterogeneity were studied by stratifying original estimates using predefined subgroup analysis by grouping geographic locations (Asia or North America), study quality (NOS score ≤ 6 or NOS score >6), adjusted for possible confounders (adjusted or unadjusted estimates). For sensitivity analysis, we excluded study that was published as abstract or provided RR as effect size for estimates. Additionally, a sensitivity analysis was performed based on simple size. To evaluate the effect of each given study on the overall risk estimate, a sensitivity analysis was also conducted by excluding one study each time. Finally, the presence of publication bias was assessed by visual inspection of the funnel plot and further evaluated with the rank correlation test of Begg and Mazumdar [72].

A two-tailed *P* <0.05 was regarded as statistically significant. The corresponding calculation and graphical visualization of forest and funnel plots were performed using Review Manager version software 5.3.5 (Nordic Cochrane Center) and Stata statistical software version 13.0 (StataCorp, USA), respectively.

## SUPPLEMENTARY MATERIALS FIGURE AND TABLES


