# Supplementary material for: Metformin therapy and the risk of colorectal adenoma in patients with type 2 diabetes: A meta-analysis
Source: Oncotarget. 2016 Nov 26;8(5):8843–53. doi: 10.18632/oncotarget.13633 (PMC5352447; doi:10.18632/oncotarget.13633)
Supplement: Supplementary file 1 [file oncotarget-08-8843-s001.pdf]

Metformin therapy and the risk of colorectal adenoma in patients with type 2 diabetes: A meta-analysis

SUPPLEMENTARY FIGURE AND TABLES

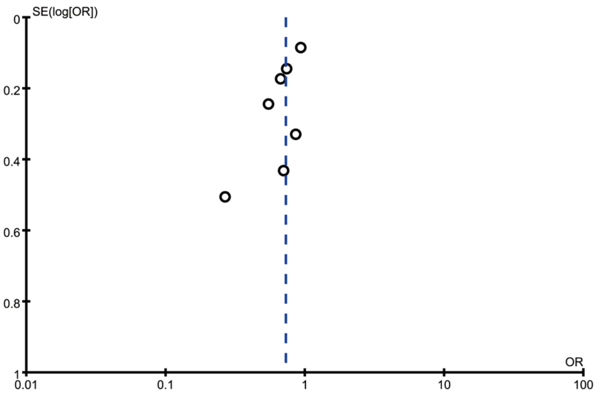

Supplementary Figure S1: Funnel plot assessing for publication. No significant publication bias was noted. OR, odds ratio; SE, standard error.

Supplementary Table S1: Newcastle-Ottawa Scale for assessment of quality of in included cohort studies

	Selection				Comparability	Outcome			
**Sub group**	**Representativeness of exposed Cohort**	**Selection of the non-exposed cohort**	**Ascertainment of exposure**	**Outcome of interest was not present at start of study**	**Study control for age/gender and additional factor**	**Assessment of outcome**	**Was follow-Up long enough for outcome to occur**	**Adequacy of follow-up of cohort**	**Overall quality score (max= 9)**
Chung et al	*	*	*	*	**	*	*	-	8
Cho et al	*	*	*	*	**	*	*	-	8
Jain et al	*	*	*	*	**	*	-	-	7
Kanadiya et al	*	*	*	*	**	*	*	-	8
Kim et al	*	*	*	*	*	*	*	-	7
Lee et al	*	*	*	-	*	*	-	*	6
Marks et al	*	*	*	-	*	*	-	*	6

Each asterisk represents if individual criterion within the subsection were fulfilled.

Supplementary Table S2: Search strategy used in PubMed

No.	Query Results	Results	Date
#1	“Colorectal Neoplasms”[Mesh]	165264	09 June 2016
#2	“colon neoplasm*” [Title/Abstract] OR “colon polyp*” [Title/Abstract] OR “colon adenoma*” [Title/Abstract] OR “large bowel neoplasm*” [Title/Abstract] OR “large bowel polyp*” [Title/Abstract] OR “large bowel adenoma*” [Title/Abstract] OR “rectum neoplasm*” [Title/Abstract] OR “rectum polyp*” [Title/Abstract] OR “rectum adenoma*” [Title/Abstract]	4578	09 June 2016
#3	#1 OR #2	166044	09 June 2016
#4	“Metformin”[Mesh]	9088	09 June 2016
#5	“Dimethylbiguanidine”[Title/Abstract] OR “Dimethylguanylguanidine” [Title/Abstract] OR “Glucophage” [Title/Abstract] OR “Metformin Hydrochloride” [Title/Abstract] OR “Hydrochloride, Metformin” [Title/Abstract] OR “Metformin HCl” [Title/Abstract] OR “HCl, Metformin”[Title/Abstract]	166608	09 June 2016
#6	#3 AND (#4 OR #5)	68	09 June 2016
